# Macon Medical Society

**Published:** 1885-01

**Authors:** 


### Society Reports.

### MACON MEDICAL SOCIETY.

Reported for THE ATLANTA MEDICAL AND SURGICAL JOURNAL.

STATED MEETING, NOVEMBER 4th, 1884.

Dr. C. H. Hall, president, in the chair.

Annual election of officers. President, Dr. J. P. Stevens; Vice-President, Dr. K. P. Moore; Secretary and Treasurer, Dr. H. J. Williams; Librarian, Dr. C. H. Hall; Corresponding Secretary, Dr. N. G. Gewinner; Reporter, Dr. H. McHatton.

After election of officers Dr. E. G. Ferguson read the essay for the evening.

### DO MENTAL SHOCKS TO THE MOTHER LEAVE IMPRESSIONS UPON THE FŒTUS IN UTERO.

How universally prevalent the idea that impressions on, or shocks to the mind of the mother, are reflected upon the fœtus in utero, is evidenced by the citation of every conceivable defect in or blemish on the body of the infant or grown man, as traceable to an exact period, at which some shock or impression was created upon the mind of the mother before the birth of her child. Discolorations (maculæ) of the skin are attributed to some object, bearing a similar appearance, having presented itself to the mother while enceinte and creating such a profound impression, and of such lasting duration, that through the subtle agency of inexplicable laws, left the impress on the fœtus as perfect as the camera of a photographer's instrument takes its copy. Unsatiated appetites for innumerable articles of food are held to be fruitful sources of these manifestations in the discolorations of the skin, called birth-marks so frequently seen on the babe at birth, from their bearing a crude resemblance to such articles as may have been denied the mother at that particular period and for which she had an unquenchable longing. Fright is one of the exciting causes most frequently accused of leaving indelible impressions, and so certain are many mothers of this, that the very moment can be given at which the impression was made, and to further authenticate their assertions, most positively contend that the response was felt by movement at that very moment, showing most conclusively to their minds that the child even experienced the shock. Mothers are not so much to be blamed, after all, for entertaining these preposterous ideas, when we find so many practitioners maintaining and encouraging the same views, to say nothing of so-called respectable medical journals, publishing accounts of eccentricities in nature, one of which I quote from the *Medical World*, published in Philadelphia, bearing date March, 1884: "Laura Savernie, known in all museums and circuses as the tattooed woman, gave birth on Thursday night, February 28th, in Baltimore, to a boy baby who bears upon his body all the marks possessed by his mother. The boy is a bouncing little fellow and both he and his mother are doing well. The latter and her husband arrived in Baltimore the week previous, their last exhibition having been at Frederick, Md., on Washington's birth-day. The mother's married name is Mrs. Adolph Morath. Dr. A. Trego Shertzer, of Baltimore, attended the woman, and was very much surprised when he found the babe's skin marked as if with India ink. Upon it are designs of flowers, leaves, an anchor and chain, an eagle, and other figures. A number of doctors of the city have been called in to see the babe and all have expressed surprise at the curious reproduction of the marks on the mother's skin."

Having always maintained the belief that impressions or shocks to the mind of the mother were not transmissible to the fœtus in utero, and reading of this extraordinary case, in a so-called respectable medical journal, I felt there must be a mistake in my conception of the anatomy of the human reproductive organs, and so wrote to Dr. A. Trego Shertzer, of Baltimore, to know if such marks were upon the child, and in reply to my note he wrote as follows:

BALTIMORE, March 17.

DEAR DOCTOR—Yours of 14th inst. just received. I delivered the tattooed woman. Her infant was not tattooed at birth, though I believe it is now being tattooed. I have put cards in several papers denouncing the fraud. Yours respectfully,

A. TREGO SHERTZER, M. D

Showing the utter fallacy of the article, and how liable to mislead the unsuspecting, and carry conviction to the minds of those who are laboring in doubt.

How does it come that medical men, at the head of medical journals, can give credence to such bare-faced physiological absurdities? We know that shock to one portion of the nervous system reflects upon other and even remote parts, but that these impressions could be conveyed to the fœtus in utero is an utter absurdity. The only connections between the fœtus and mother consists of the placenta and umbilical cord. The umbilical cord is composed of blood vessels (two arteries and one vein) to convey nourishment to and effete matter from the fœtus. No nervous communication exists. The placenta hasn't the trace of nervous connection with the mother. The fœtus floats in a bag of water, not even coming in contact with the interior of the uterus. 'Tis true, many miscarriages occur from fright or other shock to the nervous system of the mother, but these shocks are conveyed to that portion of the nervous system supplying the uterus in the mother, causing contractions and expulsion of its contents, having nothing whatever to do with the fœtus further than its expulsion. The question will doubtless be asked, "How do children inherit the characteristics of the parent if not through the nervous influence of the mother?" 'Tis more easily asked than answered. But all I can say to that is, that as an oak is produced from an acorn, having all the characteristics of the parent tree, without any nervous communication having existed between the acorn and the tree from the time it matured and fell into suitable soil to the time it grew into a tree in any way shape its form or structure, so the child inherits the characteristics of the parents. 'Tis through a Divine law, too subtle for human power to fathom.

Dr. C. H. Hall:

I agree with Dr. Ferguson. I can see no connection between mental impressions or shock and deformities or so-called mother-marks. I have never seen a case where such influence could be followed out to my own satisfaction. Have seen cases where mothers have been subjected to violent mental impressions and have examined the off-springs carefully, but could find no results. I was out in a boat with a lady who was in the second month of pregnancy, in a storm, and we had given up all hope of being saved. The idea of her pregnancy was constantly in her mind. The child at this day evidences no physical or mental effect of the strain to which the mother was exposed. Women have often gone through trying scenes and have been delivered at full term of normal children. Many of us know of the case of a celebrated Southern lady who went through one of the campaigns of Virginia from the time of conception to delivery, without any unpleasant results to the child. I know of a child born in this city, deficient in one limb. I inquired of the mother if she could trace this deficiency to any unpleasant circumstance occurring during pregnancy, but she denied anything of the kind. Sometimes mothers claim some unpleasant occurrence or sight as the cause of defects in their children. I remember a case of a lady whose son was born with two fingers and a thumb; the mother claimed that she had been annoyed by a parrot during the early months of pregnancy. Another case of a child, one-half the face being covered by a scarlet mark, the mother tracing it to the appearance of sherry-cobblers, of which she was very fond. Another of a child with six webbed fingers and toes, traced by the mother to the sight of a swimming animal. I, myself, cannot believe in such things having any influence upon the development of the fœtus in utero during the early months of pregnancy. Impressions upon the mind of the mother, and perhaps also of the father, during coitus might, possibly, affect the future of the child.

Dr. Gewinner:

I was called to deliver a lady at full term. The child had a full moon face, large staring eyes and flat nose. This lady was frightened during the third or fourth month by a mask. She fainted at the time, and was worried by the occurrence all during pregnancy. The child bore a perfect resemblance to the mask, so the father says. It was a horrible sight and only lived a few moments. I believe that there is a nervous supply to the uterus and that the uterus is similiar to a leyden-jar. Nerve-force and electricity are almost identical.

Dr. McHatton said:

I am of the opinion that impressions upon the nervous system of the mother exert no influence on the anatomical construction of the fœtus in utero, and am sure that I have some pretty good authorities with me. That a mother may bring forth a child with a blasted nervous system, after having herself gone through severe mental and physical trials, there is no question, the immense number of infants during the siege in Paris are sufficient proof.

First pregnancies influence the subsequent ones as they do in animals. This we often see in the resemblance of children of a second marriage to the first husband. Twins have been born when one was black and the other white, and when as far as could be learned there was no question of superfecundation. Still, we should be careful in drawing conclusions from animals. When a cow gives birth to twins (male and female) the female is called a free martin and is barren. In the human being the female of twins (male and female) is not barren. Our patients always try to account for their ills in a way that seems reasonable to themselves. The great drawback to most of the cases in question, is that the stage of development of the fœtus, and the time of the shock do not correspond. I have heard of one case where the sight of the stump of an arm caused a child to be born minus an arm. The stump was seen in the middle of the ninth month of pregnancy, and it has always been a question in my mind, what became of that child's arm after it dropped off.

One of our most distinguished physiologists, in his lectures, relates the case of a Roman Emperor's wife, who was chased by a Moor and through fright brought forth a Moor. I fully agree with him in his opinion that the Moor must have caught her.

Dr. Moore said:

MR. PRESIDENT—That there are many forces at work in nature, of which we know nothing, goes without saying. Darkness and obscurity has so shrouded many natural laws that we see them, even those which we partially understand, as through a glass, darkly. We sometimes get glimpses into Nature's wonderful laboratory, and feel that we partially understand the workings of her mysterious forces, when in truth we have such vague and erroneous conceptions of them as to leave us in absolute darkness. The scientific world has made wonderful progress within the last few years in unraveling the tangle-skein of Nature's mysteries. The investigations of hungry searchers after truth in the microscopic field, has recently thrown much light upon the true etiology of many diseases. Neurologists have sought a solution of many human ills in the nervous system, and have evolved much light in this direction; and the various investigation commissions in almost every department of medical science has impressed us recently with the idea that the gray dawn of a new era in the history of our profession betokens the near approach of a brighter day. A very intelligent physician told me recently that he could always tell when his wife came unwell though he might be many miles away. The same pains and general pelvic malaise, with which she suffered, came upon him; and that he had demonstrated it so often as to make it unquestionable with him, and yet he could offer no solution to the mystery.

How often has it occurred to all of us, that strange impressions would come to us, reminding us forcibly that we had seen and heard something of just the same character of that which now presents itself, yet we can form no conception of the time and place; these impressions leave us as suddenly as they came upon us, and we can not tell whence they came nor whither they have gone. The power exercised by the so-called spiritualists, and of such persons as Miss Hurst, to whom Dr. Stephens has alluded, has never been satisfactorily explained. All these mysterious things are yet veiled in darkness to us; and while we can not understand them, it does not necessarily follow that these unseen forces have no laws of their own, to which we are strangers. While I can not say that it is true that a child *can* be and is often marked by impressions made upon the mother's mind and nervous organization, yet I would not undertake to say that it *was impossible* for such impressions and such arrests of development to be made. And indeed, in view of the weight of evidence, I rather incline to the opinion that it is possible; but how it is done I leave for the future to reveal. Dr. Gewinner's case seems to be directly to the point.

Dr. Holt:

I have never given this subject serious thought before to-night, and am not therefore prepared to enter into the discussion fully. The fairest test, it seems, would be to subject a woman to hideous surroundings. Would any of you allow a woman in pregnancy to go to see a monster? On the contrary, you tell your patients to avoid disagreeable sights, and have her surroundings as pleasant as possible. I once had a patient who asked if her child was marked, and on being told that it was, said that it was a strawberry. The mark did resemble a ripe strawberry.

Dr. Stevens said:

"Because we cannot account for certain natural phenomena upon purely physiological principles, we are not warranted in positively denying the truth of such phenomena. There are certain occult forces which science has not been able clearly to demonstrate in their operation upon the human organism. The correlation of the inorganic forces, light, heat, electricity, motion, gravity, chemical force, and vital force, is recognized by all advanced scientists. In the case of Lula Hurst there is evidently a force of which we have no recognition. It is not electricity, magnetism, nor any of the acknowledged forces.

Yet who will deny the resultants of certain manual operations? The cause, the application of the open palm of the hand, is followed by consequences that demand the exercise of extraordinary antagonistic physical energy. We cannot deny the consequences, for they are patent to our senses. We are, therefore, compelled to refer them to the operation of an inexplicable, indefinable agency of which we have no rational conception, except from its consequences. Clairvoyance, table tipping, mind reading, apparitions, haunted houses, etc., have been regarded by scientists as merely the resultants of disordered imaginations, and physical aberrancy. Yet a commission of such gentlemen as Balfour Stewart, Prof. Henry Sidgwick, Archbishop Trench, and others among the foremost scientists of the age, after two years of careful investigation, research and experiment, "have reached the conclusion thet there is a formidable array of evidence in favor of such beliefs." Because we cannot trace a direct physiological relation between cause and effect, we cannot absolutely deny the connection between apparent cause and effect. A woman *enceinte* is fearfully impressed by the sight of an object abhorrent to her moral sensibilities, and her offspring bears the marks of these distinctive characteristics in the fœtus in utero. Can we deny the fact, although we cannot account for the *modus operandi* of the fact? It is patent to our sense of observation. We are obliged to concede the truth, although the *modus operandi* is hidden from our mental vision. We have analogous instances of similar phenomena among inferior animals. It is well known among stock-breeders, that if a mare is allowed to breed from an ass with her first foal, all of her subsequent foals, when bred from horses, bear, to a greater or less degree, the asinine peculiarities of the first progenitor, in elongated ears and other physiological peculiarities. This resultant is evidently attributed to the psychological impression made upon the mare previous to her first cohabitation with the male. A mare, before her first conception, is placed in intimate relationship to what is called a "teaser." An animal of this name was what is called a sorrel in color, with white legs extending to the knees on all four legs. He was also highly bred in his genealogy. She was subsequently bred to a bay horse of inferior pedigree. The colt born of this cohabitation presented the identical color-marks of the "teaser" as well as his high bred characteristics in action, spirit and endurance. Jacob took advantage of the obtuseness of his father-in-law, Laban, when he covenanted to serve him for all the cattle that were "ring-streaked and spotted, and all the she goats that were speckled and spotted, and every one that is speckled and spotted among the goats," for hire to Laban. "And

Jacob took rods of green poplar and of the hazel and chestnut tree and fitted white streaks in them, and made the white appear what was in the rods." The consequence was that by this device Jacob soon became rich in the extraordinary increase of his flocks, circumvented the penuriousness of his father-in-law, and fairly and proudly won the heart and hand of his beloved. A large preponderance of ring streaked, spotted and speckled appeared among his flocks and confounded the obtuseness of Laban.

So we cannot always account for the inscrutable and mysterious ways of nature, but are often compelled to bow to the logic of facts, and wait for future developments to reveal the rationale of hidden forces.

Dr. Ferguson, concluding the discussion, said:

How many hundreds and thousands of enceinte women are there in the world who are exposed to all sorts of disgusting and horrifying spectacles who give birth to babes perfectly formed. If it was a rule that mental shock was productive of those monstrosities, there is no doubt in my mind that nearly every woman is exposed to mental shock at some time of her pregnancy capable of producing such monstrosities as we see in every-day life, and if such was the case we would have a race of curiosities far removed from human beings. None of the inferior animals are affected by shock. Then why should the human race be more subject to shock than other animals of the animal kingdom? It is my humble judgment that any impression (if any can be conveyed) must be at the moment of coitus, otherwise, how can the mal-formed chicken be accounted for, since one hen lays the egg and another hatches it. If during the period of gestation these influences were manifest in the offspring, then why not eggs hatched in an incubator give chickens with wooden legs?

Owing to the lateness of the hour the regular subject of discussion for the evening was postponed.

Dr. McHatton reported the following case:

At our last meeting I spoke of a case of scarlatinous nephritis, in which we had no evidences of albumen. A case of grave kidney lesion has come under my observation since, which shows what errors we may make if we put too much dependence on the existence or non-existence of albumen in the urine. A patient came to my office complaining of general lassitude, poor appetite, dyspnœa on exertion, slight nausea and mouches volantes, secretion of urine about normal in quantity.

He was sixty years of age, in good flesh, but looked a little haggard. On examination I found no dropsy. The apex of the heart in the sixth intercostal space, slightly to the left of the linea mammalis. Palpation gave an enlarged area of impulse, but no thrill. He had a mitral regurgitant and an aortic direct murmur, with a feeble first sound.

His urine was a pale yellow; acid, specific gravity, 10.10, with no visible sediment. A careful examination of the specimen by heat and nitric acid, also by Heller's method, was negative. Microscopial examination gave a number of large and small hyaline with a few granular casts, many of the hyaline casts being almost invisible. I had no hesitancy in making a diagnosis of chronic interstitial nephritis, and look upon the case as one liable to an attack of uræmic coma at any time. On the other hand, he may continue moderately well for an indefinite period.

